# Advances in the analysis of complex food matrices: Species identification in surimi-based products using Next Generation Sequencing technologies

**DOI:** 10.1371/journal.pone.0185586

**Published:** 2017-10-02

**Authors:** Alice Giusti, Andrea Armani, Carmen G. Sotelo

**Affiliations:** 1 FishLab, Department of Veterinary Sciences, University of Pisa, Pisa, Italy; 2 Instituto de Investigaciones Marinas (IIM-CSIC), Vigo, Spain; Universita degli Studi di Milano-Bicocca, ITALY

## Abstract

The Next Generation Sequencing (NGS) technologies represent a turning point in the food inspection field, particularly for species identification in matrices composed of a blend of two or more species. In this study NGS technologies were applied by testing the usefulness of the Ion Torrent Personal Genome Machine (PGM) in seafood traceability. Sixteen commercial surimi samples produced both in EU and non-EU countries were analysed. Libraries were prepared using a universal primer pair able to amplify a short *16SrRNA* fragment from a wide range of fish and cephalopod species. The mislabelling rate of the samples was also evaluated. Overall, DNA from 13 families, 19 genera and 16 species of fish, and from 3 families, 3 genera and 3 species of cephalopods was found with the analysis. Samples produced in non-EU countries exhibited a higher variability in their composition. 37.5% of the surimi products were found to be mislabelled. Among them, 25% voluntary declared a species different from those identified and 25% (all produced in non-EU countries) did not report the presence of molluscs on the label, posing a potential health threat for allergic consumers. The use of vulnerable species was also proved. Although the protocol should be further optimized, PGM platform proved to be a useful tool for the analysis of complex, highly processed products.

## Introduction

Present changes in socio-demographic features and people lifestyle, particularly in developed countries, have radically shifted consumers’ eating habits and their market choices. With the general increasingly speeding lifestyles and individualisation tendencies, available time for cooking has in fact reduced, so consumers normally prefer “time saving” products as well as affordable prices. Ready-to-eat products, which do not require a further heating or processing step before consumption, have increasingly appeal consents due to their cheapness, storage easiness and attractive appearance [1]. Among products of animal origin, many processed foods can be included under the definition of ready-to-eat food, such as ham, sausages, dairy products (milk, cheese, spreads), smoked fish, prepared salads, nuggets and others.

Surimi is a stabilized myofibrillar protein compound obtained from mechanically deboned fish flesh that is repeatedly washed with water and blended with cryoprotectants [2]. This fish paste represents an intermediate product used in the preparation of a variety of ready-to-eat seafood commodities, called surimi-based products (SBPs), marketed in different forms such as sticks, slices, crumbs, lobster tails-like, etc. [3]. SBPs, originally produced, marketed and consumed in Asian countries, are increasingly appreciated worldwide, especially in North America and Europe [4]. To date, they are in fact commonly produced also by Western food processing industries. Initially, Alaska pollock (*Gadus chalcogrammus*) was the main species used for surimi production. Then, due to its overexploitation, numerous previously underutilized fish species have started to be used [2, 5–7]. Cephalopods, particularly squids, are also often used in surimi manufacture [2,4], mainly thanks to the gelation properties of their proteins or as flavouring ingredients [8]. Therefore, surimi represents a multispecies seafood product, as its production can imply the use of an extremely wide range of species [2,5]. According to the current EU law on food labelling, it is not mandatory to provide the commercial and/or scientific name of the seafood species present in SBPs [9–11], although some brands report it voluntarily (author’s note). However, the presence of ingredients potentially causing allergies, listed in the Regulation (EU) No 1169/2011 (including “fish” and “molluscs”) must be declared. As they never represent the major ingredient of SBPs, the use of cephalopods may be undeclared, causing a potential hazard for allergic consumers.

Since surimi is a highly processed product, the use of morphological characters to identify which species have been used is impossible. Thus, species identification through DNA analysis is useful to verify the information reported on the label (if the species is declared) and to detect the eventual presence of undeclared allergenic ingredients such as cephalopods. Moreover, it allows to promote the sustainable environmental management, particularly if overexploited and/or endangered fish species are used. SBPs were actually proved to be particularly involved in mislabelling cases, regardless of their origin [5–7]. However, few studies assessing the composition of these types of products have been conducted since now, as the possibility to detect species within products containing a mixture of species goes beyond the capability of the analytical techniques routinely applied in food control. Available studies applied DNA-based techniques involving a classical Sanger-based DNA sequencing phase, such as FINS [7] as well as DNA-Barcoding [6]. However, Sanger-based DNA sequencing alone has often been considered a not feasible approach for a complete description of species composition in mixed food analysis. Galal-Khallaf et al. [5] have recently highlighted the necessity to appeal to more suitable techniques for these products, combining the classical direct sequencing of PCR products with a PCR cloning technique with subsequent plasmid sequencing. PCR cloning, even though effective, is a rather laborious and time-consuming approach to be routinely used in laboratories. In this regard, a metagenomics approach, using High Throughput Sequencing technologies, commonly known as Next Generation Sequencing (NGS), represents a useful alternative to PCR cloning to identify species in highly processed multispecies products. This technique is faster and even more informative than cloning, since it can detect also low-represented species in mixtures [12]. Because of that, NGS results attractive for food inspection research field, even though they cannot yet be considered enough mature to be applied as routine method. More studies aimed at improving its accuracy as well as correcting their error sources are needed [13]. Preliminary studies were performed on artificial mixtures of meat species to verify the method’s robustness [14,15] and the NGS have been practically applied to commercial products in the work of Muñoz-Colmenero et al. [16], that detected the animal species contained in candies, that were selected as a model of highly processed foods. As regards seafood, to the best of our knowledge, NGS technologies have been applied in commercial fish cakes [17], as well as in highly processed cod products [18]. In addition, Kappel et al. [19] tested their effectiveness in discriminating tuna species within experimental mixtures. Given the scarce available literature, more studies focused in optimizing NGS protocols and testing the potentiality of these technologies in seafood analysis are undoubtedly required. In fact, although the still quite high costs, NGS prices are progressively dropping during the years, so that, in a near future, these techniques could be routinely applied to this research field.

A preliminary study, conducted with the aim to help in the preparation phase of NGS libraries, showed the ability of the primer’s pair developed by Chapela et al. [20] to amplify a short fragment of mitochondrial 16S ribosomal gene (*16SrRNA*) in many fish and cephalopod species used in surimi production [21]. In the present study, we used for the first time the Ion Torrent Personal Genome Machine (PGM) to apply a metabarcoding approach to the analysis of the composition of some surimi-based products (SBPs) purchased on international market. The primer’s pair of Chapela et al. [20] was used for amplifying the DNA fragment to be turned into standard libraries. This study aimed at providing an analytical starting point to better approach such new techniques for their future application to a wider range of multispecies seafood products.

## Materials and methods

### Samples collection and DNA extraction

Sixteen SBPs were collected (Table 1). Among them, fourteen were purchased from Spanish and Italian grocery stores and two were collected from imports coming from third countries by the staff of the Border Inspection Post (BIP) of Leghorn (Italy). All the samples were stored at -20°C before DNA extraction. Total DNA was extracted from all the SBPs with the protocol proposed by Armani et al. [22], starting from 100 mg of tissue, and quantified using a Qubit^™^ 3.0 Fluorometer (Thermo Fisher Scientific).

**Table 1:** Commercial SBPs analysed in this study.

Sample (IC)	Collection site	Producer country	Commercial denomination/product description	Ingredients	Declared species
SUR-1	Big distribution (Spain)	Spain	Surimi product—Sticks	Surimi (**fish**), water, sunflower oil, **cephalopod** (mollusc), starch and modified starch, salt, egg albumin, vegetable protein, flavor (containing shellfish), flavor enhancer (monosodium glutamate), sugar, natural dyes (cochineal and paprika extract)	ND
SUR-2	Big distribution (Spain)	Germany	Cooled surimi product	Surimi (**Fish**), water, **cephalopod** (mollusc), sunflower oil, starch modified, wheat flour, soy protein, sea salt, vegetable protein, egg albumen, flavor (contains crustaceans) and cephalopod ink (mollusc)	ND
SUR-3	Big distribution (Spain)	Spain	Grated surimi product	Surimi (**fish**), water, sunflower oil, **cephalopod** (mollusc), starch and modified starch, salt, egg albumin, vegetable protein, flavor (containing crustacea), flavor enhancer (monosodium glutamate), sugar, natural colorings (cochineal and paprika extract)	ND
SUR-4	Big distribution (Spain)	Spain	Sticks	Surimi 47% [**fish** and **cephalopods** (molluscs)], water, corn starch, modified starches (gluten), sunflower oil, aroma and crab extract [crustaceans, soy flavor enhancer (monosodium glutamate, E635)], salt, egg, vegetable protein (gluten), coloring (paprika extract)	ND
SUR-5	Big distribution (Spain)	Spain	Surimi slices	Surimi 49% (**fish**), water, rice starch, sunflower oil, crab aroma and **extract** [crustaceans, **molluscs**, soy flavor enhancers (monosodium glutamate, E 635)], egg albumin, protein vegetable (gluten), modified starch (gluten), salt, sugar, preservative (potassium sorbate, coloring (paprika extract)	ND
SUR-6	Big distribution (Spain)	France	Surimi duo–lobster flavour	38% Surimi (**Fish pulp** 92% -***Micromesistius poutassou***, sugar, trehalose, stabilizers: sorbitols, E450, E451), water, wheat starch (contains gluten), rehydrated egg powder, canola oil, salt, flavorings (contains gluten, fish and shellfish), sugar, flavor enhancer: monosodium glutamate; coloring: paprika extract	*Micromesistius poutassou*
SUR-7	Big distribution (Spain)	Spain	Surimi sticks–seafood flavour	Surimi (**Fish**), water, starch and modified starch, **cephalopod** (mollusc), sunflower oil, egg whites, vegetable protein, salt, flavor enhancer (E621), flavor (contains crustaceans), sugar, coloring (E120 and E160c)	ND
SUR-8	Big distribution (Spain)	Spain	Surimi sticks	Surimi (**Fish**), water, sunflower oil, **cephalopod** (mollusc), starch and modified starch, crab extracto (crustacean), salt, egg whites, vegetable protein, flavor enhancer (monosodium glutamate), white wine extract, sugar, natural colorings (cochineal and paprika extract)	ND
SUR-9	Big distribution (Spain)	Spain	Processed seafood product with garlic	**Fish protein**, water, sunflower oil, wheat flour, **cephalopod** (mollusc), olive oil, salt, soy protein, vegetable protein, milk protein, egg white, aromas, flavor enhancer (glutamate monosodium), stabilizer (xanthan gum), acidity (lactic acid), cephalopod ink, (mollusc), natural extracts (garlic and chilli). May contain traces of crustacea.	ND
SUR-10	Big distribution (Spain)	Spain	Surimi sticks–seafood flavour	Surimi (**Fish**), water, sunflower oil, **cephalopod** (mollusc), starch and modified starch, salt, egg white, vegetable protein, flavorings (contain crustaceans), flavor enhancer (monosodium glutamate), sugar, natural colorings (cochineal and paprika extract)	ND
SUR-11	Big distribution (Spain)	Spain	Surimi product	Surimi (**Fish**), water, sunflower oil, wheat flour, **cephalopod** (mollusc), salt, soy protein, vegetable protein, milk protein, egg albumen, aromas, flavor enhancer (E621), stabilizer (E415), acidity regulator (E270), cephalopod ink (mollusc)	ND
SUR-12	Big distribution (Spain)	Spain	Surimi in brine–crab flavour	**White fish**, **squid**, sugar, sorbitol (E420), polyphosphates (E452), water, potato starch, crabmeat (4%), vegetable oil, egg, salt, flavoring crab, flavor enhancers (E621, E635), stabilizer (carrageenan), colorants (E171, E120, E160c). Pickle ingredients: water, salt, citric acid, trisodium citrate, sodium benzoate, tartaric acid	ND
SUR-13	Small retailers (Italy)	Korea	Lobster tails imitation	**Alaska pollock** (55,78%), water, wheat starch, egg white, lobster extract, rice alcohol, salt, sugar, soybean oil, natural coloring: paprika	*Gadus chalcogrammus*
SUR-14	Small retailers (Italy)	Lithuania	Surimi sticks–crab flavour	Surimi (38%), water, starch, egg white, rapeseed oil, soy proteins, salt, sugar, tapioca starch and acetylated potato, crab aroma, whole egg, E621, E631, E635, E160, E120	ND
SUR-15	BIP (Italy)	China	Surimi sticks–crab flavour	Surimi 44%, wheat starch, soybean oil, crab flavor, crab extract, egg white, potato flour	*Nemipterus sp*.
SUR-16	BIP (Italy)	Thailand	Fish-shaped crab meat imitation	Surimi 35%, palm oil, modified tapioca starch, egg white, soy protein, crab extract.	*Nemipterus sp*.

IC: Internal code BIP: Border Inspection Post

### DNA amplification and purification

The DNA samples were amplified with the primer pairs 16sf-var 5´-CAAATTACGCTGTTATCCCTATGG-3´ and 16sr-var 5´-GACGAGAAGACCCTAATGAGCTTT-3´ designed by Chapela et al. [20] using illustra^™^ puReTaq Ready-To-Go^™^ PCR Beads (GE Healthcare). For each tube containing the bead, 2 μl of 200 nM of each primer, 1 μl of 50 ng of template DNA and nuclease-free water (Life Technologies) were added, for a final reaction volume of 25 μl. DNA was amplified on an Applied Biosystems Veriti^™^ 96 well Thermal Cycler (Thermo Fisher Scientific) with the following cycling program: denaturation at 94°C for 3 min; 35 cycles at 94°C for 40 s, 60°C for 40 s, and 72°C for 40 s; final extension at 72°C for 7 min. 5 μL of each PCR product was checked by electrophoresis on a 2% agarose gel and the presence of fragments of the expected length was assessed by a comparison with the standard marker O'GeneRuler DNA Ladder (Thermo Fisher Scientific). Double-stranded PCR products were purified with Agencourt^®^ AMPure^®^ XP Kit for DNA purification (Beckman Coulter, Beverly, Massachusetts, USA) on a DynaMag^™^-2 magnet magnetic rack (Thermo Fisher Scientific) following the procedure proposed by the manufacturer. Purified PCR products were quantified using a Qubit^™^ 3.0 Fluorometer (Thermo Fisher Scientific).

### Preparation of barcoded libraries

A specific barcoded library was prepared for the amplicon obtained from each SBPs using the Ion Plus Fragment Library Kit (Thermo Fisher Scientific) (IPFL kit), that allowed amplicons’ end-repair and ligation to Ion-compatible adapters.

#### Amplicons end-repair and purification

20 ng of each amplified sample were diluted in a total volume of 79 μl of nuclease-free water (Life Technologies). Amplicons’ end-repair was done by adding 20 μl of 5X End Repair Buffer and 1 μl of End Repair Enzyme (both provided by the IPFL kit) and incubating the reaction at room temperature for 20 minutes. The samples were then purified with Agencourt^®^ AMPure^®^ XP Kit for DNA purification (Beckman Coulter, Beverly, Massachusetts, USA) on a DynaMag^™^-2 magnet magnetic rack (Thermo Fisher Scientific) following the procedure proposed by the manufacturer.

#### Adaptors ligation, nick reparation and purification of the amplicons

Adaptors provided in the Ion Xpress^™^ Barcode Adapters 1–16 (Thermo Fisher Scientific) were used. The same Ion Xpress^™^ P1 Adapter was ligated to the amplicons obtained from all the SBPs samples whereas a unique Ion Xpress^™^ Barcode Adapter for each sample was used. Adaptors ligation and nick repair phases were done in a final reaction volume of 100 μl, containing: 25 μl of end-repaired and purified amplicon with 10 μl of 10X Ligase Buffer, 2 μl of dNTP Mix, 2 μl of DNA ligase and 8 μl of Nick Repair Polymerase (all provided by the IPFL kit), 2 μl of Ion P1 Adapter, 2 μl of Ion Xpress^™^ Barcode Adapter, 49 μl of nuclease-free water (Life Technologies). Each reaction mix tube was run on an Applied Biosystems Veriti^™^ 96 well Thermal Cycler (Thermo Fisher Scientific) with the program proposed by the IPFL kit manufacturer. The samples were then purified with Agencourt^®^ AMPure^®^ XP Kit for DNA purification (Beckman Coulter, Beverly, Massachusetts, USA) on a DynaMag^™^-2 magnet magnetic rack (Thermo Fisher Scientific) following the procedure proposed by the manufacturer. Purified products were quantified by an Agilent 2100 Bioanalyzer (Agilent Genomics).

#### Libraries amplification and quantification

Libraries were amplified on an Applied Biosystems Veriti^™^ 96 well Thermal Cycler (Thermo Fisher Scientific) in a total reaction volume of 130 μl, containing 100 μl of Platinum^®^PCR SuperMix High Fidelity, 5 μl of Library Amplification Primer Mix (both provided by the IPFL kit) and 25 μl of unamplified library. The cycling program suggested on the IPFL kit protocol was applied. Amplified libraries were purified with Agencourt^®^ AMPure^®^ XP Kit for DNA purification (Beckman Coulter, Beverly, Massachusetts, USA) on a DynaMag^™^-2 magnet magnetic rack (Thermo Fisher Scientific) following the procedure proposed by the manufacturer. Agilent 2100 Bioanalyzer (Agilent Genomics) was used to determine the molar concentration of each barcoded library. Three equimolar pools of barcoded libraries were prepared: barcoded libraries from SUR-1 to SUR-6 (Pool 1), from SUR-7 to SUR-12 (Pool 2) and from SUR-13 to SUR-16 (Pool 3) were pooled together. The three pools were quantified on Agilent 2100 Bioanalyzer (Agilent Genomics) or Library TaqManTM Quantitation Kit (Thermo Fisher Scientific) following the procedure proposed by the manufacturer, and then diluted as proposed by the Ion PGM^™^ Hi-Q^™^ Chef Kit (Thermo Fisher Scientific).

### Massive DNA clonal parallel amplification and sequencing by synthesis

#### Ion Sphere^™^ Particles (ISP) preparation and chips loading

The Ion PGM^™^ Hi-Q^™^ Chef Kit (Thermo Fisher Scientific) was utilized to prepare template-positive Ion Sphere^™^ Particles (ISP) and to load three Ion 314^™^ v2 BC sequencing chips (Chip 1 for Pool 1, Chip 2 for Pool 2 and Chip 3 for Pool 3) on Ion Chef^™^ System (Thermo Fisher Scientific) following the manufacturer protocol.

#### Sequencing by synthesis

The three chips sequencing was done on an Ion PGM^™^System (Thermo Fisher Scientific) using the Ion PGM^™^ Hi-Q^™^ Sequencing Kit (Thermo Fisher Scientific) according to the manufacturer protocol. Reads obtained from the three sequencing chips were processed by the software Torrent Suite^™^ version 5.0 (Thermo Fisher Scientific).

### Bioinformatics analysis

#### Data quality assessment

Each sequencing chip was primarily overall evaluated with the Torrent Suite^™^ version 5.0 (Thermo Fisher Scientific) software on the basis of the final number of usable reads (overall quality assessment) and of the reads length. Regarding the overall quality, we considered as acceptable a ISP loading higher than 70%, jointly with a polyclonal amount lower than 20% and a final percentage of usable library higher than 80% (weak presence of low quality reads). The reads length was considered as cornerstone of a good sequencing outcome if the distribution of the major part of the reads (expressed as a graphic peak) corresponded to the length of the target amplicon. Then, the FASTQ files for each barcoded sample (that contain the raw sequences and their quality values) were downloaded from the software and analysed through the program FastQC High Throughput Sequence QC Report version 0.11.5 (www.bioinformatics.babraham.ac.uk/projects/). We put attention to the total number of the raw reads and to their length (both provided by the FastQC program), that might be long enough to include the target amplicon.

#### Reads taxonomic assignment and analysis of frequencies

The raw FASTQ files were sent to Era7 Bioinformatics (Cambridge MA, USA) for obtaining their taxonomic profile. In details, according to the final report providing from Era7, the sequences were filtered on the basis of a minimum length of 100 bp and a maximum of ~300 bp (to contain the target amplicon). The sequences were also filtered on the basis of their quality in order to ensure a highly supported taxonomic assignment. Filtered reads were then assigned to a taxonomic tree node based on sequence similarity to 16SrRNA genes included in Era7 internal database, built with 16S sequences extracted from from RNAcentral database (http://rnacentral.org/). RNAcentral database includes rRNAs from a wide set of important databases as SILVA, GreenGenes, RDP, RefSeq and ENA. The NCBI taxonomy was used and for taxonomic assignment the MG7 method, that is based on a BLAST comparison of each read against the 16S ribosomal RNA database, was applied. Samples’ species identification was based on the BLAST results. In particular, taxonomic assignment was done using 2 different algorithms: (i) Best BLAST Hit (BBH) assignment, obtained by the BLASTN of each read against the internal 16S database (each read was assigned to the taxon corresponding to the Best Blast Hit over a threshold of similarity) and (ii) Lowest Common Ancestor (LCA) assignment, where each read was assigned to the most probable taxon where it could come from. The frequencies for each taxonomy node were also assessed. Phylum, Family, Genus and species distribution in all the samples was assessed. The frequencies were expressed in % with respect to the total merged reads of each sample. Moreover, BBH assignments were used to calculate the diversity index for each sample. In particular, Simpson’s diversity index was applied.

### SBPs mislabelling assessing

A preliminary analysis of the information reported on the label was performed in the light of the current European legislation [9,10] and coupled with the analytical results to evaluate the mislabelling degree of the SBPs. In particular, we used the following criteria to consider one case of mislabelling:: (A) labels did not report the precise term “fish” among the ingredients (B) labels did not report the precise term “molluscs” among the ingredients (whereas declared); (C) among the labels voluntarily reporting the scientific name, those in which the declared species did not correspond to the ones retrieved by the analysis; (D) labels did not declare the presence of molluscs but the analysis proved the presence of species belonging to this Phylum.

## Results and discussion

### Samples collection

The traditional SBPs trade, based on Chinese exports to Europe, has recently slowed down since Europe has increased its own SBPs manufacturing activity. Spain plays an important role in the European surimi sector, being one of the most important producer and consumer of SBPs, jointly with France [4]. Apart from the two SBPs collected at the BIP (SUR-15 and SUR-16), produced in China and Thailand respectively, the fourteen samples directly purchased in this study in Spain and Italy were produced in European countries, except for one (SUR-13) which was produced in Korea and purchased at an Italian small retailer. Twelve samples reported on the label the presence of “fish” in the list of ingredients, sometimes adding an adjective or a noun such as “white fish”, “fish pulp” or “fish protein”. The scientific name of the utilized species was reported in four samples and corresponded to *Micromesistius poutassou* (SUR-6), *Gadus chalcogrammus* (SUR-13) and *Nemipterus* spp. (SUR-15 and SUR-16). Ten samples reported the presence of “molluscs”, either among the main ingredients or as aroma/extract, or both. The percentage of surimi paste was also reported in seven samples. In all the SBPs miscellaneous ingredients were also listed, such as wheat starch, potato, salt, soybean oil and sugar (Table 1).

### Primers selection

Since the maximum target read length of Ion PGM sequencing system is 400 bp, the amplicon selected as target must be shorter. The primer pair used in this study, already tested in a previous study [20], was proved able to amplify a fragment of ~250–260 bp (depending on the species) from fish as well as a fragment of ~190–200 bp (depending on the species) from cephalopod species. Therefore, it can be successfully used for the amplification of extremely processed products such as surimi, where a high degree of DNA degradation is known [7]. In addition, although they have been originally designed to amplify only cephalopod species [20], a recent study [21] has shown their capability in amplifying DNA from more than eighty species of fish and cephalopods.

### High Throughput Sequencing and data analysis

#### Overall quality control of the reads

Modern high throughput sequencers can generate tens of millions of sequences in a single run. Therefore, preliminary suitable quality control checks of the raw data are required before approaching subsequent analysis.

Overall, 674.983 raw sequences (78% of total analysed) and 674.826 raw sequences (73% of total analysed) were obtained from Chip 1 and Chip 2, respectively. The sequences were considered as “usable” since they respected the threshold values established in materials and methods section (see paragraph “Data quality assessment”). Moreover, the global distribution peak of the reads corresponded to the length of the target amplicon. Chip 3 was less performant as the 668.081 raw sequences evaluated as usable represented only 58% of all the sequences analysed. Moreover, the polyclonal percentage (32%) was higher than the threshold value. However, since the final usable library was good (85%), few low-quality sequences were present (14%) and the read lengths corresponded to the expected length of the target amplicons, these data were considered as usable for subsequent bioinformatics analysis.

#### FastQC analysis

Comparison between FastQC analysis of raw and filtered reads per each sample was reported in Table 2. Raw reads considered suitable in term of quality by Torrent Suite^™^ version 5.0 software (Thermo Fisher Scientific) ranked between 2431 and 259531 and their length ranked between a minimum of 25 bp to a maximum of 354 bp. Filtration phase reduced the number of reads by selecting a minimum length of 100 bp, while the maximum length was between 262 bp and 321 bp. This step allowed to preserve the two target amplicons (250–260 bp for fish and 190–200 for cephalopods) and easing the taxonomic analysis by removing too short fragments that could have given uncertain results. After the filtration step, the total number of usable reads was not much lower than that of raw reads, with a preservation ranging from 75.3% to 88.2%, in the case of Chip 1 and Chip 2. On the contrary, final usable reads of Chip 3 were lower, with a preservation ranging from 37.8% to 51.1%, confirming the worse outputs obtained in the previous overall reads analysis.

**Table 2:** Comparison between FastQC analysis of raw and filtered reads obtained from each sample.

Sample	Raw reads		Filtered reads		Preserved reads (%)
	Number	Length (bp)	Number	Length (bp)	
SUR-1	21597	25–333	19056	100–313	88.2
SUR-2	79174	25–332	68051	100–299	86
SUR-3	69682	25–292	59820	100–278	85.8
SUR-4	154476	25–325	126906	100–310	82.2
SUR-5	184772	25–346	155618	100–294	84.2
SUR-6	106418	25–354	91054	100–311	85.6
SUR-7	68584	25–312	53647	100–290	78.2
SUR-8	2431	25–262	1831	100–262	75.3
SUR-9	72162	25–318	59494	100–292	82.4
SUR-10	82491	25–321	64473	100–321	78.1
SUR-11	259531	25–319	207017	100–302	79.8
SUR-12	115899	25–339	96113	100–299	82.9
SUR-13	54134	25–307	23853	100–277	44.1
SUR-14	54572	25–297	28104	100–292	51.5
SUR-15	38203	25–296	17976	100–293	47.1
SUR-16	57674	25–316	21815	100–300	37.8

#### Reads taxonomic assignment, analysis of frequencies and samples diversity index

**Fig 1:**
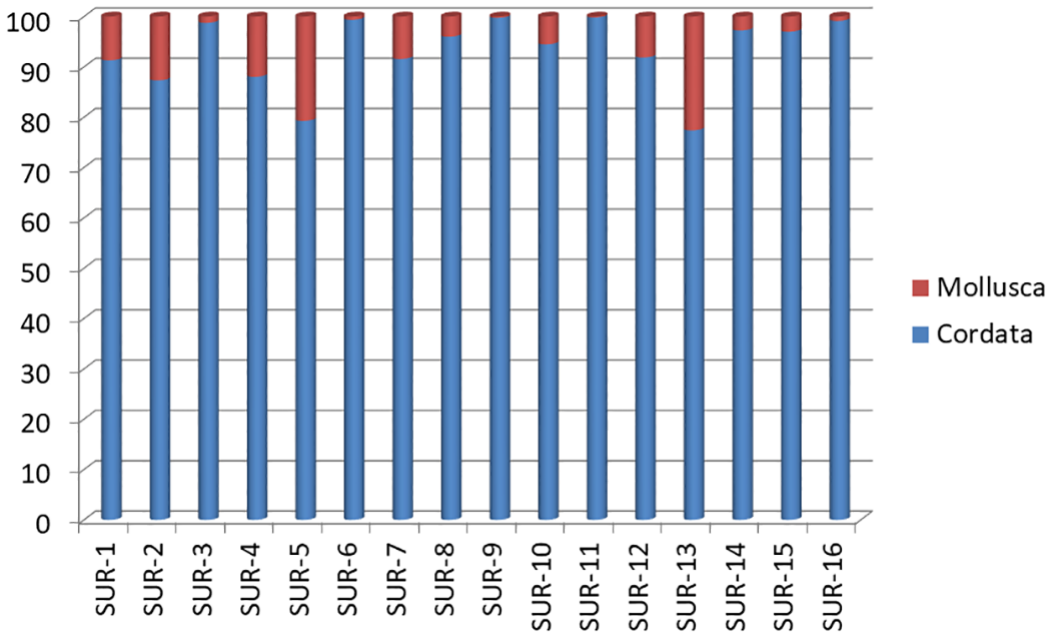
Phylum distribution (cumulative % LCA) in each analysed SBP.

Phylum distribution (cumulative % LCA) in the samples is shown in Fig 1. Even though, as predictable, the Phylum Cordata was the most represented in all the SBPs (always greater than 75% and in 75% of the samples greater than 90%), the Phylum Mollusca was also found in 100% of the samples, in different percentages. In particular, 25% of the samples contained less than 1% of molluscs’ DNA, 50% between 1% and 10%, 12.5% between 10% and 20%, and 12.5% more than 20%. Regardless of the quantities of DNA found in the samples, these results substantially confirmed that the use of molluscs in surimi products is very common in the seafood industry. Literature reported in fact the high resistance to freeze-induced denaturation and to proteolytic attack of cephalopods myofibrillar proteins [2], as well as the fact that even a small amount of these proteins considerably improves the texture of a gel product, making it more elastic and with a greater cohesiveness [23].

**Fig 2:**
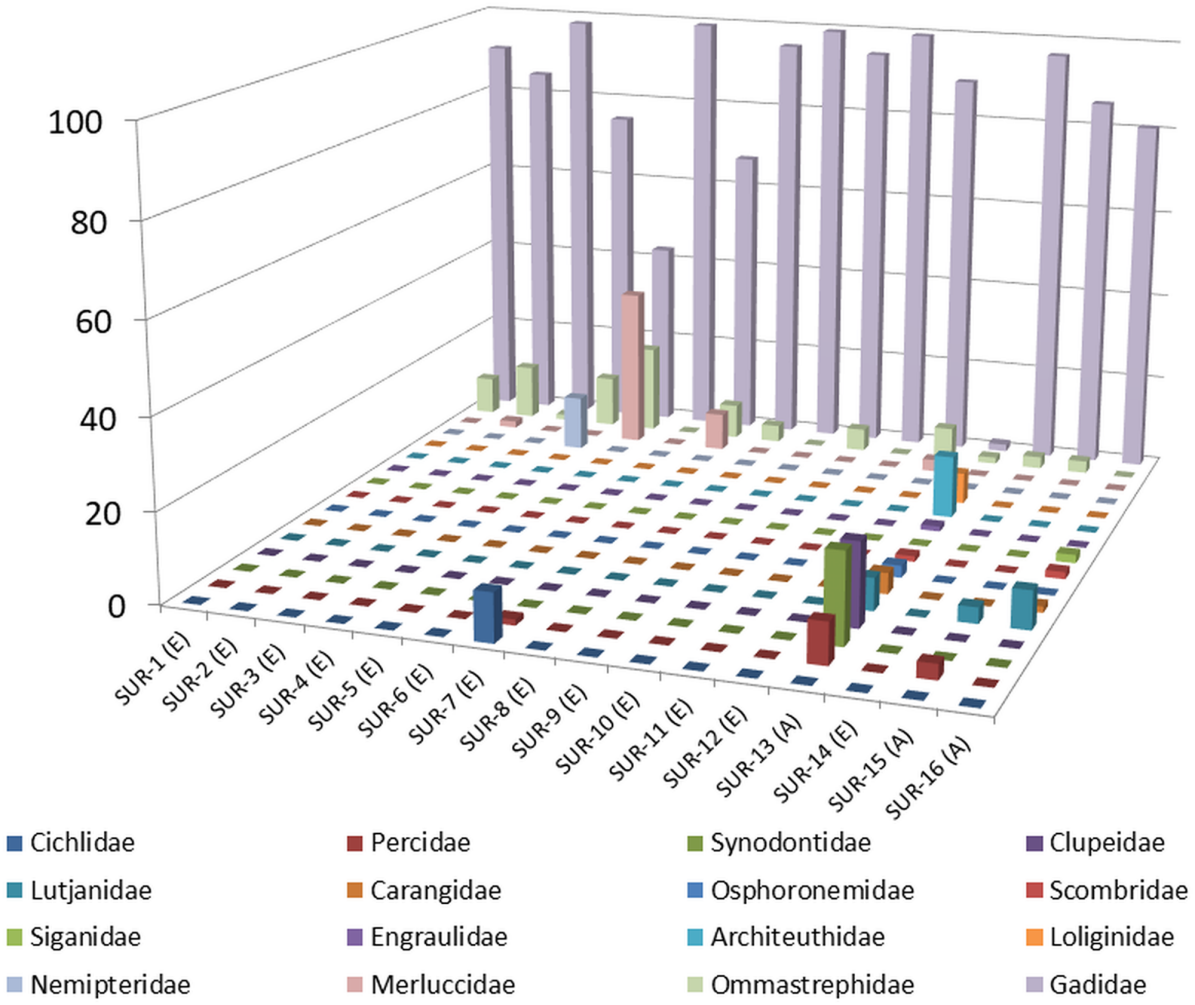
Families presence and distribution in each sample. (E): SBP produced in Europe: (A): SBP produced in Asia.

**Fig 3:**
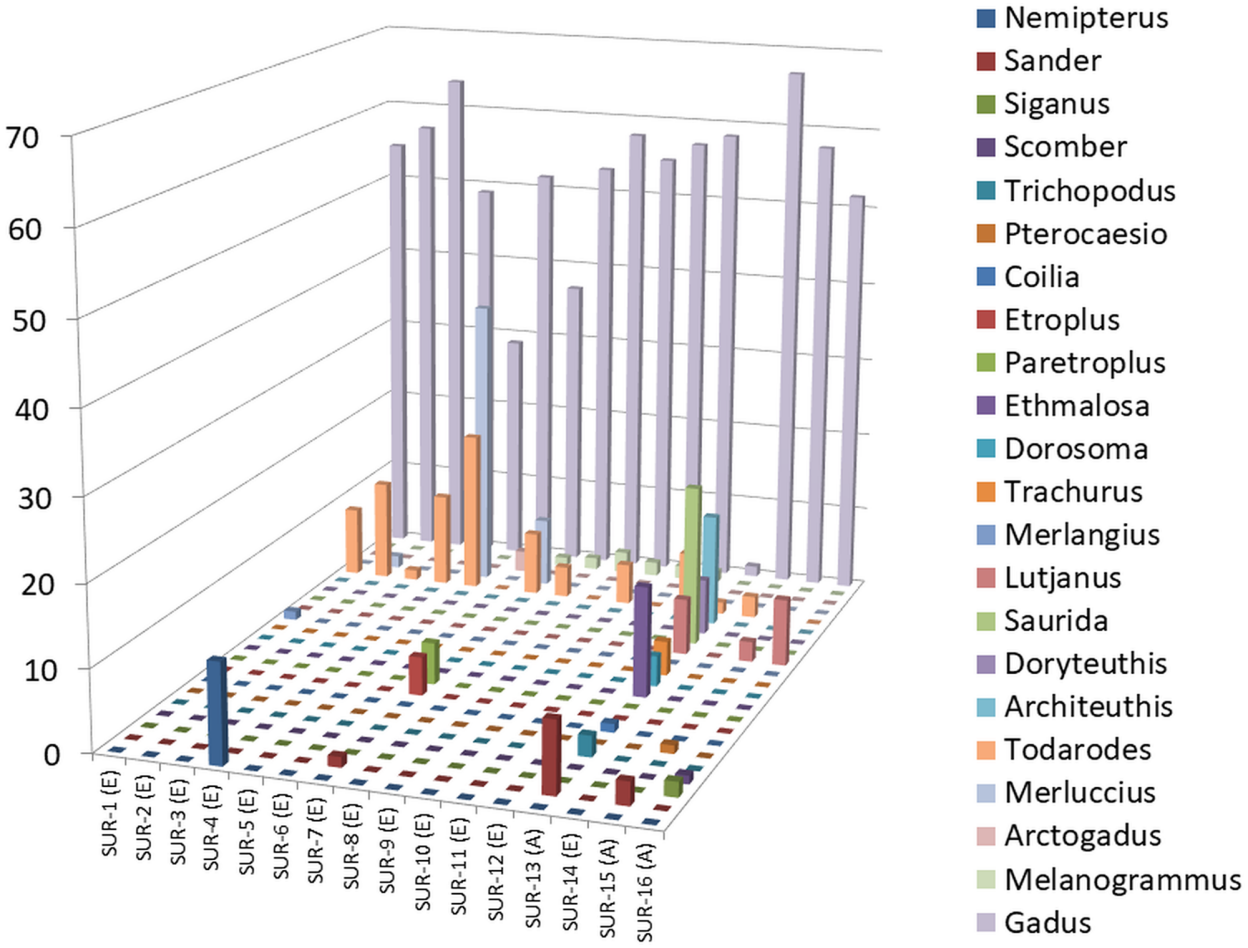
Genus presence and distribution in each sample. (E): SBP produced in Europe: (A): SBP produced in Asia.

Results of the family, genus and species distribution in all the samples were reported in Table 3. Given the great amount of assigned reads, only taxonomical entities present in the samples in amount >1% were reported. Overall, DNA from 13 families, 19 genera and 16 species of fish, and from 3 families, 3 genera and 3 species of cephalopods was found in SBPs. Figs 2 and 3 depict the distribution of families and genera in the samples. Regarding fish, although some differences in composition between EU and Asian SBPs subsisted, DNA belonging to the Gadidae family was found in 100% of the samples. The percentage was rather high in most of the cases, exceeding 90% in 50% of the samples, ranging from 70 to 90% in 31.2% of the samples and from 40 to 70% in 12.5% of the samples. Gadidae were poorly represented (<2%) only in one Asian sample (SUR-13). DNA from *Gadus* genus was found in 100% of the samples, with the species *Gadus chalcogrammus* identified in 93.75% of the samples, whereas DNA from *Gadus morhua* was detected in two samples. Also, DNA from *Arctogadus* genus/*Arctogadus glacialis* species, *Melanogrammus* genus/*Melanogrammus aeglefinus* species and *Merlangius* genus/*Merlangius merlangus* species was detected in 6.25%, 43.75% and 6.25% of the samples, respectively. DNA belonging to Merluccidae family was found in 25% of the samples, in variable percentages (from ≃ 1% to over 36%). and only *Merluccius* genus/*Merluccius merluccius* species was present in that samples. 12.3% of DNA from Nemipteridae family/*Nemipterus* genus (species identification was not reached) was found only in SUR-4. Variable percentage of DNA belonging to Carangidae (*Trachurus* spp.), Synodontidae (*Saurida undosquamis*), Clupeidae (*Dorosoma petenense* and *Ethmalosa fimabriata*), Percidae (*Sander* spp.), Engraulidae (*Coilia grayii*) Caesionidae (*Pterocaesio tile*), Siganidae (*Siganus* spp.), Lutjanidae (*Lutjanus bengalensis* and *Lutjanus rivulatus*) and also to freshwater families such as Osphoronemidae (*Trichopodus leeri*) and Cichlidae (*Etroplus maculatus* and *Paretroplus maculatus*) was found in some samples. It is important to underline that in all the samples a variable percentage of DNA not identifiable at species level, but only at family or genus level, was present (Table 3). These results substantially confirmed those already reported in literature. In fact, the most part of the fish species found in the samples were those commonly used for surimi production or sometimes reported in studies aimed at identifying species in such type of products. Exceptions were represented by the non-EU samples SUR-13, SUR-15 and SUR-16 and by the EU sample SUR-7, in which unconventionally species, never used until now, were found. Regarding molluscs, it is primarily important to reiterate the fact that, although DNA from molluscs was detected in all the samples, taxonomical assignment was reported in Table 3 only if its presence was higher than 1%. DNA from Ommastrephidae family/*Todarodes* genus/*Todarodes pacificus* species was found in 75% of the samples. DNA belonging to Loliginidae family/*Doryteuthis* genus/ *Doryteuthis opalescens* species and Architeuthidae family/*Architeuthis* genus/*Architeuthis dux* species was also found in one sample (SUR-13). Differently from fish, the molluscs species found in the analysed samples did not correspond to those listed as the most used in surimi manufacture. In fact, the species *T*. *pacificus*, which was the most representative in this study, was very rarely reported, while the species *A*. *dux* was never reported at all.

**Table 3:** Family, genus and species distribution in all the samples with relative percentages.

Sample		Family	Genus	Species
SUR-1	Cordata	Gadidae 91.2%	*Gadus* 54.5% *Merlangius* 1.1%	*Gadus chalcogrammus* 54.2% Gadidae 34.9% *Merlangius merlangus* 1.1%
	Mollusca	Ommastrephidae 8.6%	*Todarodes* 8.6%	*Todarodes pacificus* 8.6%
SUR-2	Cordata	Gadidae 85.1%Merluccidae 1.5%	*Gadus* 57.1%*Merluccius* 1.1%	*Gadus chalcogrammus* 56.9% Gadidae 27.0% *Merluccius merluccius* 1.5%
	Mollusca	Ommastrephidae 12.5%	*Todarodes* 12.5%	*Todarodes pacificus* 12.5%
SUR-3	Cordata	Gadidae 98.2%	*Gadus* 63.5%	*Gadus chalcogrammus* 63.20%Gadidae 33.6%
	Mollusca	Ommastrephidae 1.2%	*Todarodes* 1.2%	*Todarodes pacificus* 1.2%
SUR-4	Cordata	Gadidae 75.0% Nemipteridae 12.3%	*Gadus* 49.1% *Nemipterus* 12.3%	*Gadus morhua* 25.5% Gadidae 25.0% *Gadus chalcogrammus* 19.7% *Nemipterus spp*. 12.3% *Gadus spp*. 3.8%
	Mollusca	Ommastrephidae 11.7%	*Todarodes* 11.7%	*Todarodes pacificus* 11.7%
SUR-5	Cordata	Gadidae 42.9% Merluccidae 36.3%	*Merluccius* 36.3% *Gadus* 28.9%	*Merluccius merluccius* 36.3% *Gadus chalcogrammus* 28.8% Gadidae 13.5%
	Mollusca	Ommastrephidae 20.1%	*Todarodes* 20.1%	*Todarodes pacificus* 20.1%
SUR-6	Cordata	Gadidae 99.3%	*Gadus* 51.6% *Arctogadus* 2.7% *Melanogrammus* 1.3%	Gadidae 43.2% *Gadus morhua* 41.9% *Gadus spp*. 9.1% *Arctogadus glacialis* 2.7% *Melanogrammus aeglefinus* 1.3%
SUR-7	Cordata	Gadidae 67.2% Cichlidae 10.7% Merluccidae 8.5% Percidae 1.3%	*Gadus* 36.9% *Merluccius* 8.6% *Paretroplus* 5.1% *Etroplus* 4.7% *Sander* 1.3% *Melanogrammus* 1.1%	*Gadus chalcogrammus* 36.7% Gadidae 28.9% *Merluccius merluccius* 8.5% *Paretroplus maculatus* 5.1% *Etroplus maculatus* 4.7% *Sander spp*. 1.3% *Melanogrammus aeglefinus* 1.1%
	Mollusca	Ommastrephidae 7.9%	*Todarodes* 7.9%	*Todarodes pacificus* 7.9%
SUR-8	Cordata	Gadidae 95.3%	*Gadus* 53.1% *Melanogrammus* 1.5%	*Gadus chalcogrammus* 52.4% Gadidae 39.7% *Melanogrammus aeglefinus* 1.5%
	Mollusca	Ommastrephidae 3.9%	*Todarodes* 3.9%	*Todarodes pacificus* 3.9%
SUR-9	Cordata	Gadidae 99.4%	*Gadus* 57.1% *Melanogrammus* 2.7%	*Gadus chalcogrammus* 56.7% Gadidae 39.3% *Melanogrammus aeglefinus* 2.7%
SUR-10	Cordata	Gadidae 94.4%	*Gadus* 55.8% *Melanogrammus* 1.7%	*Gadus chalcogrammus* 54.4% Gadidae 37.6% *Melanogrammus aeglefinus* 1.7%
	Mollusca	Ommastrephidae 5.1%	*Todarodes* 5.1%	*Todarodes pacificus* 5.1%
SUR-11	Cordata	Gadidae 99.4%	*Gadus* 57.1% *Melanogrammus* 1.6%	*Gadus chalcogrammus* 56.2% Gadidae 40.2% *Melanogrammus aeglefinus* 1.6%
SUR-12	Cordata	Gadidae 89.1% Merluccidae 2.7%	*Gadus* 58.5% *Merluccius* 2.7% *Melanogrammus* 1.4%	*Gadus chalcogrammus* 58.1% Gadidae 29.0% *Merluccius merluccius* 2.7% *Melanogrammus aeglefinus* 1.4%
	Mollusca	Ommastrephidae 7.4%	*Todarodes* 7.4%	*Todarodes pacificus* 7.4%
SUR-13	Cordata	Synodontidae 19.9% Clupeidae 18.4% Percidae 9.1% Lutjanidae 7.1% Carangidae 4.6% Osphoronemidae 2.7% Gadidae 1.6% Scombridae 1.2% Engraulidae 1.1%	*Saurida* 19.9% *Ethmalosa* 13.6% *Sander* 8.8% *Lutjanus* 6.9% *Trachurus* 4.2% *Dorosoma* 3.8% *Trichopodus* 2.6% *Gadus* 1.3% *Coilia* 1.1%	*Saurida undosquamis* 19.9% *Ethmalosa fimbriata* 13.6% *Sander spp*. 8.8% Percomorphaceae 4.8% *Trachurus spp*. 4.2% *Lutjanus spp*. 4.1% *Dorosoma petenense* 3.6% *Lutjanus rivulatus* 2.3% *Trichopodus spp*. 1.3% *Gadus chalcogrammus* 1.3% *Trichopodus leeri* 1.2% *Coilia grayii* 1.0%
	Mollusca	Architeuthidae 13.9% Loliginidae 6.9% Ommastrephidae 1.5%	*Architeuthis* 13.9% *Doryteuthis* 6.9% *Todarodes* 1.5%	*Architeuthis dux* 13.9% *Doryteuthis opalescens* 6.9% *Todarodes pacificus* 1.5%
SUR-14	Cordata	Gadidae 96.4%	*Gadus* 66.9%	*Gadus chalcogrammus* 66.3% Gadidae 28.2%
	Mollusca	Ommastrephidae 2.7%	*Todarodes* 2.7%	*Todarodes pacificus* 2.7%
SUR-15	Cordata	Gadidae 85.8% Lutjanidae 3.5% Percidae 3.4%	*Gadus* 57.7% *Sander* 2.9% *Lutjanus* 2.5% *Pterocaesio* 1.0%	*Gadus chalcogrammus* 55.5% Gadidae 26.9% *Sander spp*. 2.9% *Gadus morhua* 1.8% *Lutjanus bengalensis* 1.1% *Lutjanus rivulatus* 1.0% *Pterocaesio tile* 1.0%
	Mollusca	Ommastrephidae 2.8%	*Todarodes* 2.8%	*Todarodes pacificus* 2.8%
SUR-16	Cordata	Gadidae 80.8% Lutjanidae 8.5%	*Gadus* 51.7% *Lutjanus* 8.3% *Siganus* 1.8% *Scomber* 1.0%	*Gadus chalcogrammus* 48.7% Gadidae 27.3% *Lutjanus bengalensis* 5.8% *Gadus morhua* 2.4% *Lutjanus rivulatus* 2.1% *Siganus spp*. 1.8%

**Fig 4:**
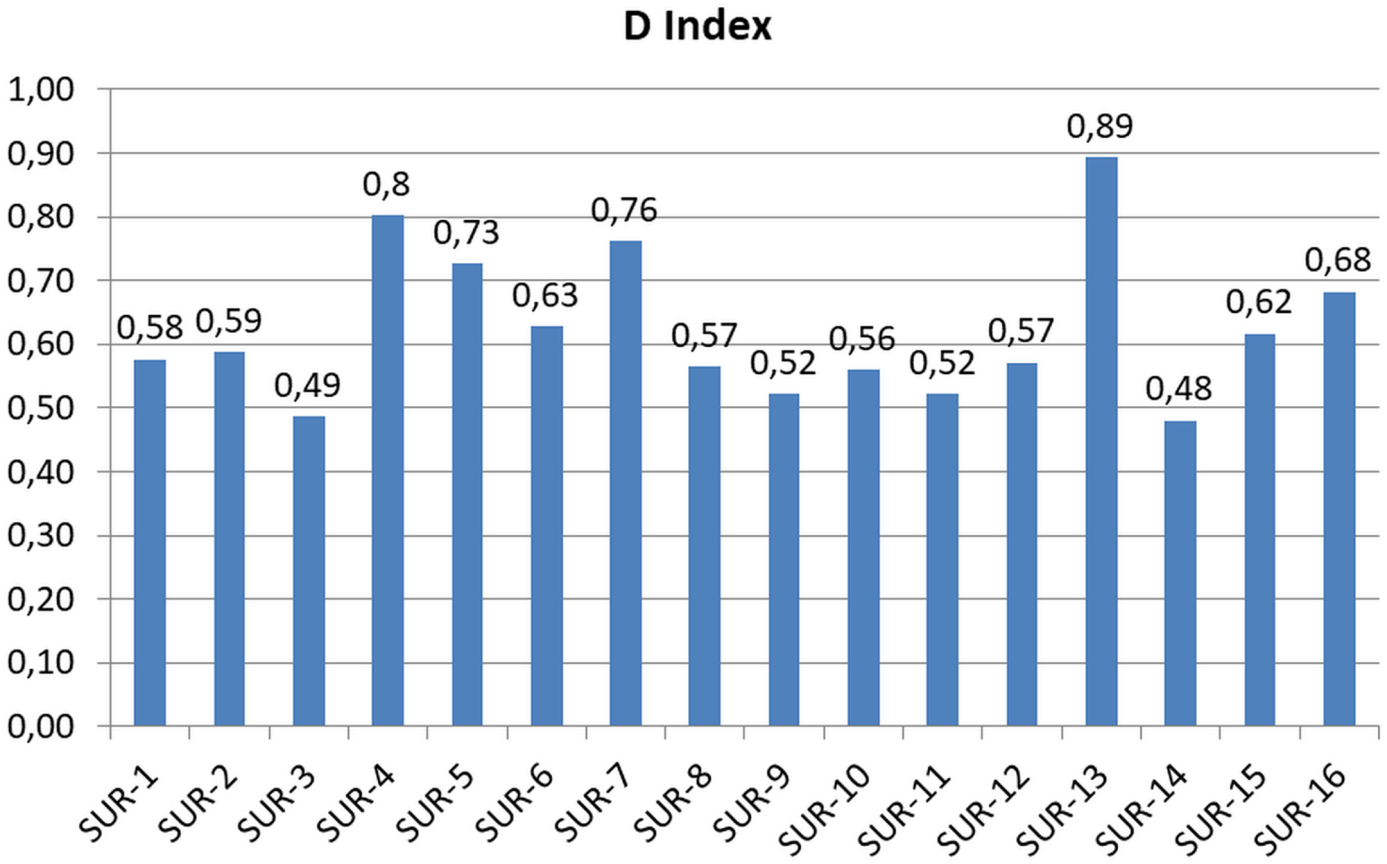
Simpson’s diversity index (D Index) values obtained for each sample.

Diversity index was calculated to reflect how many different species there were in each sample. The obtained values of the Simpson’s diversity index (D Index) were reported and graphically illustrated in Fig 4. The most part of the samples (43.7%) presented a diversity index between 0.5 and 0.6. The highest diversity index values (≥0.8) were reached by the samples SUR-4 and SUR-13, whereas the lowest (≤0.5) by the samples SUR-3 and SUR-14. The diversity index value did not seem to be directly correlated to the country origin of the sample.

### SBPs’ label information: Mislabelling assessment

Mislabelling evaluation results were reported in Table 4. Overall, 37.5% of the SBPs’ were found as mislabelled. Non-compliance involved 100% of SBPs produced in non-EU countries and 23.1% of SBPs produced in EU countries. The final mislabelling percentage was: (i) 25% of the samples did not report the exact term “fish”, but were simply labelled as “surimi” or, in the case of SUR-13, only declared the commercial denomination of the species (“Alaska pollock”) jointly with the scientific name (*Gadus chalcogrammus*). The use of the term “surimi” alone, as well as the species declaration alone, are both non-compliant with the current Regulation since it could be unclear, for the average consumer, that surimi is often produced from fish and molluscs. The presence of allergens such as fish or mollusc should be clearly indicated in the label since allergic consumers might not be aware of what they are really buying; (ii) 12,5% of the samples reported an altered form of the term “molluscs”, indicating “shellfish” or “squid”, which could be equally misleading and unclear for consumers; (iii) 25% of the samples voluntary declared a species that actually not corresponded to that found through the DNA analysis. In SUR-6, where the presence of *Micromesistius poutassou* was declared, no DNA of this species was found. On the contrary, the most part of DNA found in this sample belonged to the species *Gadus morhua* or, in lower amount, *Melanogrammus aeglefinus* and *Arctogadus glacialis*. Species substitution also involved the Asian sample SUR-13, where the declared *Gadus calchogrammus* was actually a mixture of species mostly belonging to Synodontidae, Clupeidae and Lutjanidae families, as well as SUR-15 and SUR-16, where the reported *Nemipterus* spp. was not found at all; (iv) 25% of the samples (all produced in non-EU countries) not reported at all the presence of molluscs on the label, despite mollusc DNA was found through our molecular analyses (Fig 1).

**Table 4:** SBPs mislabelling cases evaluated through both labels information analysis and sequences data results.

Sample	Label information (ingredients)		Label conformity
	Fish	Molluscs	
SUR-1	fish	- cephalopod (molluscs); - flavour (containing shellfish)	correct
SUR-2	surimi (fish)	- cephalopod (mollusc): - containing cephalopod ink	correct
SUR-3	surimi (fish)	cephalopod (mollusc)	correct
SUR-4	surimi [fish and cephalopods (molluscs)]		correct
SUR-5	surimi (fish)	extract (molluscs)	correct
SUR-6	surimi (fish pulp *Micromesistius poutassou*)	Flavorings (contains shellfish)	mislabelled B; C
SUR-7	surimi (fish)	cephalopod (molluscs)	correct
SUR-8	surimi (fish)	cephalopod (molluscs)	correct
SUR-9	fish protein	- cephalopod (molluscs); - cephalopod ink (molluscs)	correct
SUR-10	surimi (fish)	cephalopod (molluscs)	correct
SUR-11	surimi (fish)	- cephalopod (molluscs); - cephalopod ink (molluscs)	correct
SUR-12	white fish	Squid	mislabelled B
SUR-13	Alaska pollack *Gadus chalchogrammus*	NR	mislabelled A; C; D
SUR-14	surimi	NR	mislabelled A; D
SUR-15	surimi *Nemipterus spp*.	NR	mislabelled A; C^(^*^)^; D
SUR-16	surimi *Nemipterus spp*.	NR	mislabelled A; C^(^*^)^; D

NR: not reported; A: not reporting the precise term “fish” among the ingredients; B: not reporting the precise term “molluscs” among the ingredients (whereas declared); C: the voluntarily declared species not correspond to that retrieved by the analysis; D: label not declare the presence of molluscs but the analysis proved the presence of species belonging to this Phylum;

^(^*^)^: the declared species is present but in lower amount respect to other undeclared species.

### Mislabelling and health implications

In parallel with the global increase of seafood consumption, seafood allergy incidence has considerably risen over the past 40 years. To date, fish is considered one of the eight most common allergenic foods, collectively considered to be responsible for about 90% of food allergic reactions [24]. Symptomatology can be severe and sometimes fatal. Fish meat is in fact one of the foods most commonly responsible of severe anaphylaxis [25]. Parvalbumin, which is found in all fish species, is reported to be the major fish allergen for 95% of patients suffering from IgE-mediated fish allergy [26]. It is resistant to boiling and other high temperature processing, so that adverse reactions can also occur after consuming fish in processed form. Moreover, many other allergens have been recently characterised and identified. Noteworthy, there is evidence that the allergenic power of different fish species may differ to some extent, with e.g. hake and cod reportedly being among the more allergenic [24]. Molluscs hypersensitivity also represents a very common food allergy type. Consumption of molluscs is assumed to be responsible of reactions ranging from a mild oral allergy syndrome to severe symptoms such as anaphylactic shock in sensitive consumers. Tropomyosin (TM) was the first allergen identified, but also in this case other allergens have recently been characterized [27]. Allergy to surimi has been verified in a patient who reacted to 1 g of surimi [28]. According to the current food European regulation, labels must inform consumers on the allergic hazard of all seafood types [9]. To make a clearer and standardized labelling system, in terms of allergenic seafood, the terms “fish” and “molluscs” must be reported. This obligation is absolutely opportune since the presence of fish and molluscs, even in small percentage, could represent a hazard in allergic consumers. In fact, current clinical, epidemiological and experimental data do not allow determining safe allergen threshold levels that would not trigger adverse reactions in a sensitised consumer [24]. However, deficiencies in food labelling, particularly in products imported from Asian countries, have been often ascertained [29]. Therefore, the mislabelling rate involving the SBPs produced in Asian countries further confirmed the lower safety degree of these products, above all considering the absence of a standardized system for seafood labelling and traceability [30]. In this study, samples not reporting the presence of “fish” and/or “molluscs” on the label actually represent an health hazard for allergic consumers. Noteworthy, another possible health hazard is represented by the presence of the species *L*. *rivulatus* in two Asian samples, which has been reported to be associated to ciguatera poisoning (fishbase.org).

### Species composition of SBPs and evaluation of environmental impact

If the main large-scale source for surimi production is the Alaska pollock (*Gadus chalcogrammus*), the variability of fish stocks and the limitation of this species around the world has led to the exploitation of many other species, both among fish and cephalopods [2]. To date, more than 80 species are in fact reported as a resource for surimi industry [21], belonging to a wide and diverse taxonomic range. A recent study conducted by Galal-Khallaf et al. [5] has already reported the use of a wide range of new species, sometimes vulnerable, in surimi production, highlighting the necessity to proper identify species in such kind of foodstuff to better manage overexploited and/or endangered marine resources. The results of our study further confirm the presence of species traditionally used in surimi production, such as those belonging to the groups of cods, haddocks and hakes. Alaska pollock (*G*. *chalcogrammus*), and in general species included in the *Gadus* genus, was almost always found in both European and Asian SBPs. Haddock (*M*. *aeglefinus*), already reported as commonly used, was in the same way often found in European samples. On the contrary, to the best of our knowledge, whiting (*M*. *merlangus*) and Arctic cod (*A*. *glacialis*) were not reported yet as a resource for surimi production. As these two species were found in the samples produced in European countries, their presence could be explained by the fact that their habitat mostly includes the Northeast Atlantic area, so that they are probably caught in European waters and then processed within the Community. The same occurs for the European hake (*Merluccius merluccius*), which has never been reported in surimi production, while its congeners are worldwide exploited for this purpose. On the contrary, *Nemipterus* spp., already reported as widely utilized in Asiatic products [5], was found in the samples produced in the EU. Even though the identification at species level was not reached, all Nemipteridae habit tropical and sub-tropical Indo-West Pacific waters (fishbase.org). This implies that European surimi industry not only uses its own country resources, but also extra-EU species (which could be imported or directly caught in extra-EU waters by European vessels). Moreover, since surimi can be processed from the flesh of fish not only in land-based operations, but also on-board processing ships, it is not to consider improbable that European ships operate in extra-EU waters, so that the products are consequently composed by extra-EU resources.

Other species already reported in the literature were found in the analysed Asian samples, such as *Saurida undosquamis* (Brushtooth lizardfish) and *Trachurus* spp. Noteworthy, the presence of new unexpected species, generally characterized by low/not fishery interest, was detected in both European and Asian samples. Among Clupeidae, *Dorosoma petenense*, a species habiting North and Central America waters, and *Ethmalosa fimbriata*, from Eastern Central Atlantic Ocean, were found in a sample produced in Korea, together with the Indo-Pacific *Coilia grayii* (Engraulidae). *Sander* spp. (Percidae) was found in both European and Chinese samples, but unfortunately the identification at species level was not reached, so that it was not possible to attribute an origin to this fish. The Indo-Pacific *Ptaerocaesio tile* (Caesionidae) and *Siganus* spp. (Siganidae) (which is commonly as used ornamental fish) were found in samples produced in China and Thailand, respectively. Bengal snapper (*Lutjanus bengalensis*) and Blupperlip snapper (*Lutjanus rivulatus*), habiting the Indian Ocean, were found in three Asian samples. Finally, also freshwater fish were found: *Etroplus maculatus*, and *Paretroplus maculatus* (Cichlidae), habiting Indian and African waters respectively, were found in a sample produced in Europe. *P*. *maculatus*, in particular, is reported as Critically Endangered (CE) species by the IUCN Red List of Threatened Species (www.iucnredlist.org). Similarly, the freshwater species *Trichopodus leeri* (Osphoronemidae), native of Malay Peninsula, Thailand and Indonesian waters, which was found in a Korean sample, is reported as Near Threatened (NT) by the IUCN Red List of Threatened Species (www.iucnredlist.org). Among molluscs, whereas *Doryteuthis opalescens* (Ommastrephidae) has already been reported for surimi production, to the best of our knowledge no literature data concerning the use *Todarodes pacificus* (Ommastrephidae) and *Architeuthis dux* (Architeuthidae) have been reported yet.

## Conclusions

In this work, the Ion Torrent NGS technology was tested for the first time on surimi-based products. Although further studies aimed at optimizing and standardizing the analytical protocol, deepening the aspect of DNA quantification and obviously applying this technology to a larger number of samples are required, the metabarcoding approach was proved to be suitable for species identification of processed multispecies seafood products. Overall our results suggest that surimi production is not related to a definite resource, but mostly depends to the catching and processing area, fishing season and species availability. In particular, it seems that in Asian region the overexploitation enforces the use of almost all of the catch for seafood industry, often challenging the sustainable management and conservation of marine resources. In fact, given the low percentage of DNA present in the SBPs for some species, it can be excluded that these species are deliberately caught to be processed by the seafood industry but rather they occasionally enter in the production chain. However, such a great and diversified range of species, also including vulnerable and potentially toxic ones, as well as the often-undeclared presence of potentially allergic risks, strongly remarks the still too weak control system and poorly eco-friendly management around the global seafood sector, highlighting the necessity to further strengthen the tools aimed at ensuring both consumers and environmental protection.
